# Supplementary figures and images for: Nutritional Genomic Approach for Improving Grain Protein Content in Wheat
Source: Foods. 2023 Mar 25;12(7):1399. doi: 10.3390/foods12071399 (PMC10093644; doi:10.3390/foods12071399)

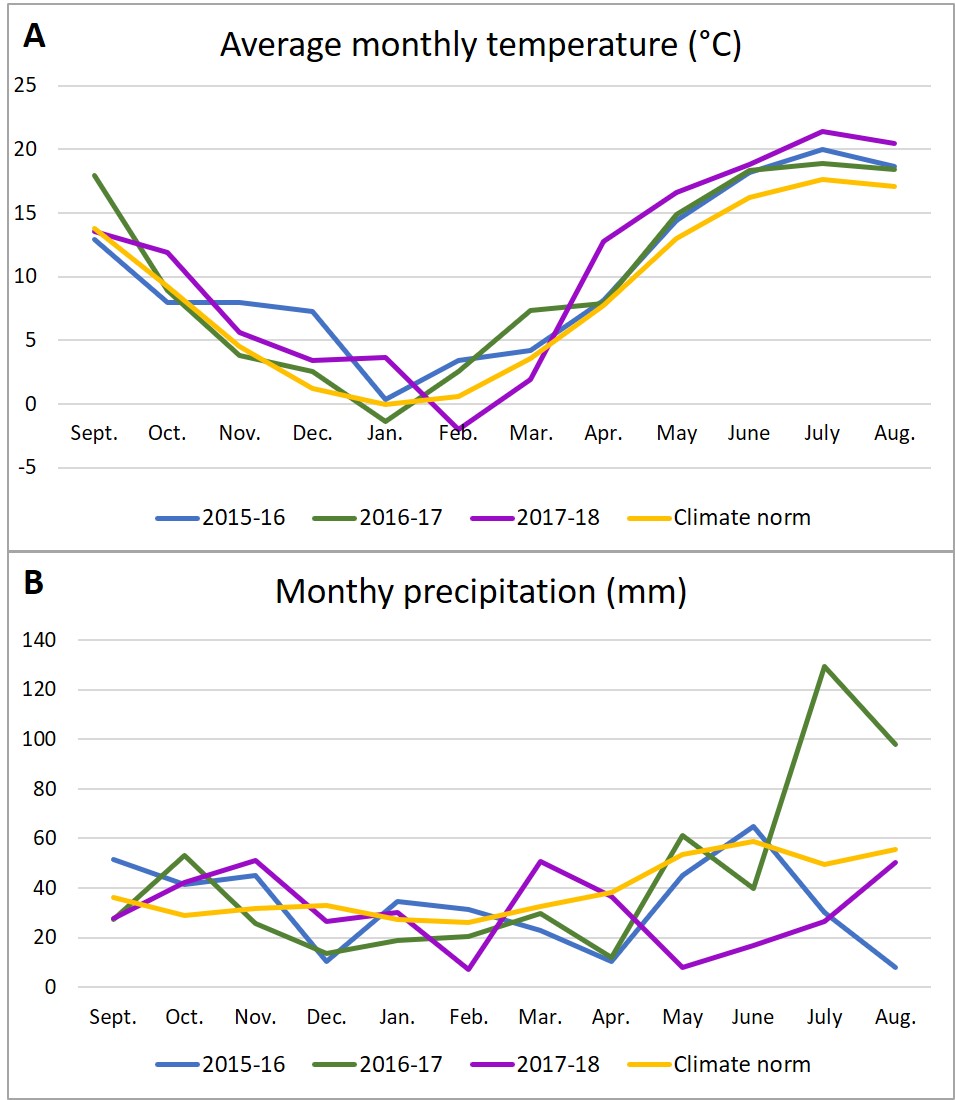

Supplement: Supplementary file 1 [file foods-12-01399-s001.zip › Figure_S1_Meteodata+Norm.jpg]

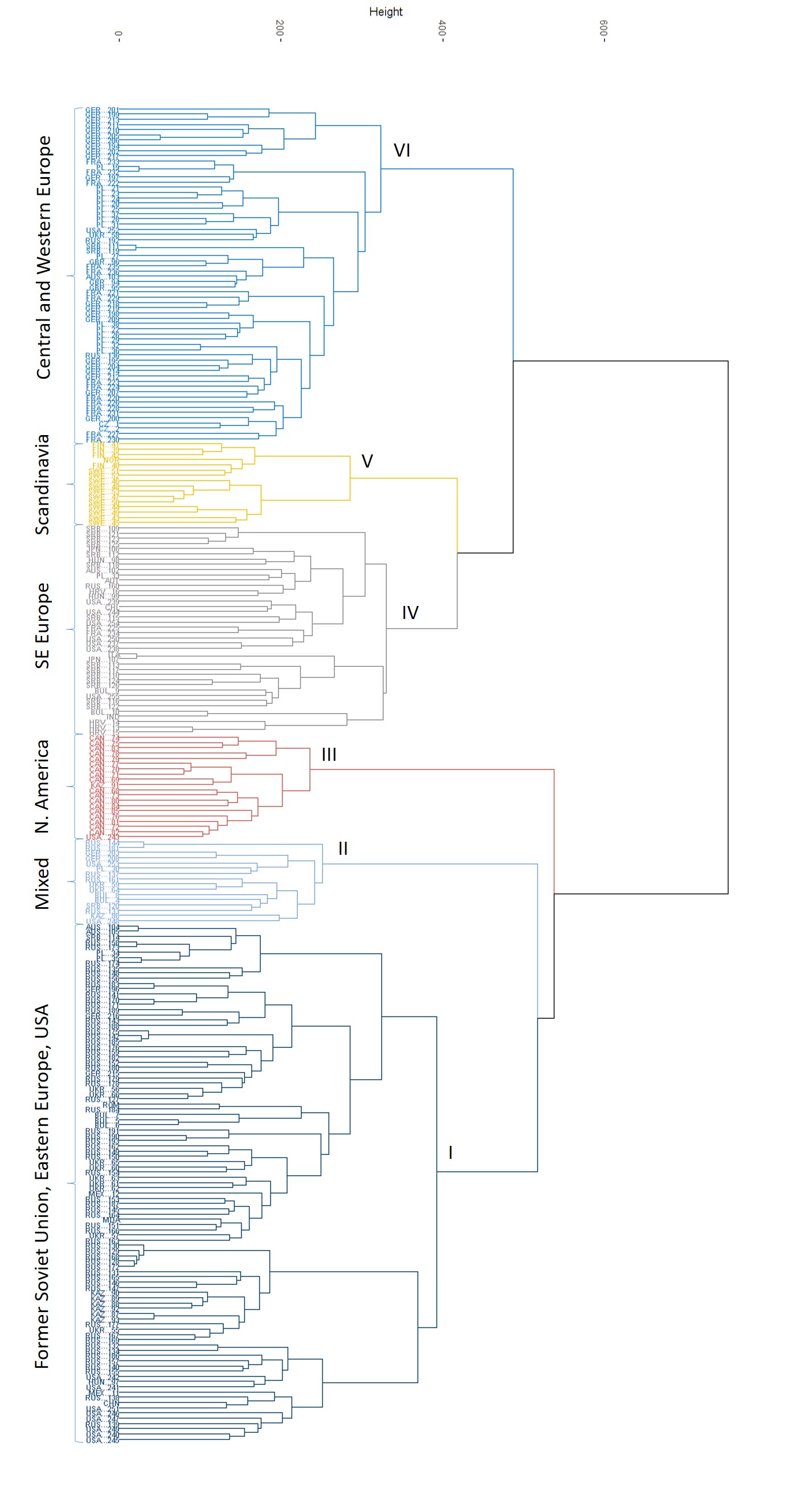

Supplement: Supplementary file 1 [file foods-12-01399-s001.zip › Figure_S2_Phylogeny_Origin.jpg]
